# Application of 3D printing surgical training models in the preoperative assessment of robot-assisted partial nephrectomy

**DOI:** 10.1186/s12893-024-02456-6

**Published:** 2024-05-28

**Authors:** Zheng Wang, Xin Yu Wang, Xiao fen Yu

**Affiliations:** 1 Cancer Center, Zhejiang Provincial People’s Hospital ( Affiliated People’s Hospital of Hangzhou Medical College), Hangzhou, 310014 China; 2 Department of Rehabilitation Medicine, Zhejiang Shuren University Affiliated Shulan Hangzhou Hospital, Hangzhou, 310022 Zhejiang China; 3 Department of the Operating Room, Zhejiang Provincial People’s Hospital, (Affiliated People’s Hospital of Hangzhou Medical College), Hangzhou, 310014 China

**Keywords:** 3D printing model, Audio-visual media, Robotic-assisted laparoscopic surgery, Partial nephrectomy, Preoperative visit

## Abstract

**Background:**

To explore the application effect of 3D printing surgical training models in the preoperative assessment of robot-assisted partial nephrectomy.

**Methods:**

Eighty patients who underwent robot-assisted partial nephrectomy surgery between January 2022 and December 2023 were selected and divided into two groups according to the chronological order. The control group (*n* = 40) received preoperative assessment with verbal and video education from January 2022 to December 2022, while the observation group (*n* = 40) received preoperative assessment with 3D printing surgical training models combined with verbal and video education from January 2023 to December 2023. The preoperative anxiety, information demand score, and surgical awareness were compared between the two groups. The physiological stress indicators, including interleukin-6 (IL-6), angiotensin II (AT II), adrenocorticotropic hormone (ACTH), cortisol (Cor), mean arterial pressure (MAP), and heart rate (HR), were also measured at different time points before and after surgery.They were 6:00 am on the day before surgery (T0), 6:00 am on the day of the operation (T1), 6:00 am on the first day after the operation (T2), and 6:00 am on the third day after the operation (T3).The preparation rate before surgery was compared between the two groups.

**Results:**

The anxiety and surgical information demand scores were lower in the observation group than in the control group before anesthesia induction, and the difference was statistically significant (*P* < 0.001). Both groups had lower scores before anesthesia induction than before preoperative assessment, and the difference was statistically significant (*P* < 0.05). The physiological stress indicators at T1 time points were lower in the observation group than in the control group, and the difference was statistically significant (*P* < 0.05). The overall means of the physiological stress indicators differed significantly between the two groups (*P* < 0.001). Compared with the T0 time point, the T1, T2, and T3 time points in both groups were significantly lower, and the difference was statistically significant (*P* < 0.05). The surgical awareness and preparation rate before surgery were higher in the observation group than in the control group, and the difference was statistically significant (*P* < 0.05).

**Conclusion:**

The preoperative assessment mode using 3D printing surgical training models combined with verbal and video education can effectively reduce the psychological and physiological stress responses of surgical patients, improve their surgical awareness, and enhance the preparation rate before surgery.

 The EndoWrist simulation robotic arm, 3D high-definition imaging system, tremor filtering, and more ergonomic design of the robotic surgery system have improved the precision of surgical operations and promoted the development of minimally invasive surgery. Since Gettman et al [1]. performed the first da Vinci robot-assisted laparoscopic partial nephrectomy (RPN), this procedure has gradually become the mainstream treatment for renal cell carcinoma [2]. The use of robotic surgery systems as operative tools is a new emerging technology in our province. Patients lack understanding of robotic surgery, which increases their doubts about the surgery, fear of disease, surgical trauma, and creates strong psychological and physiological stress reactions, affecting patient compliance and prognosis. Effective preoperative visits can help alleviate patients’ negative emotions such as nervousness, anxiety, and fear, and enable them to actively accept and cooperate with surgical treatment in the best state of mind, reduce postoperative complications, and promote rapid recovery [3, 4]. With the development of multimedia and Internet technology, the form of oral explanation combined with video visit is gradually popularized, but most of the patients and their families who do not have a medical background can only understand a part of the disease and surgical plan through video and images, and the effect of the visit is limited [5, 6]. Therefore, from January to December 2023, during preoperative visits, our robotic surgery nursing team used the 3D printed surgical training kidney models (Fig. 1) as visual aids to educate patients about the RPN procedure, in combination with verbal education and robotic surgery video interviews. We achieved good results, as described in this report.

**Fig. 1:**
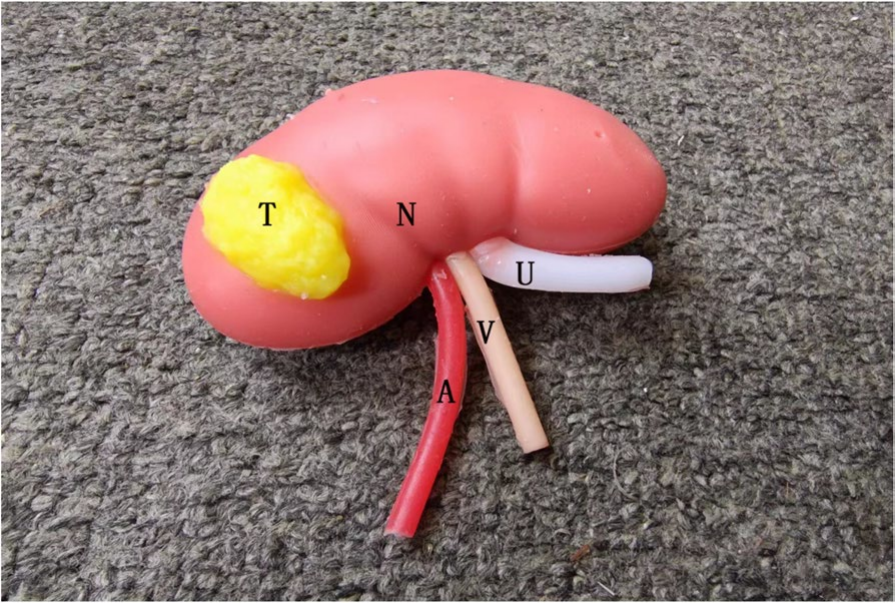
3D printed surgical training kidney models. T: tumor tissue, N:normal tissue of the kidney, A:renal artery, V:renal veins, U:ureter

## Information and methods

### Clinical information

This study was a prospective study, approved and adopted by the Ethics Committee of our hospital (Ethics No. QT 2,023,232). A total of 80 patients who underwent robot-assisted partial nephrectomy in a tertiary hospital in Hangzhou between January 2022 and December 2023were included in this study. The patients were divided into two groups according to the time sequence of their surgery: the control group from January 2022 to December 2022, and the observation group from January 2023 to December 2023. All patients included in the study were voluntary participants who signed written informed consent forms. The inclusion criteria were: (1) a highly suspected malignant kidney tumor based on the patient’s medical history and auxiliary examinations, and subsequent robot-assisted partial nephrectomy with a pathologic diagnosis of malignant kidney tumor; (2) aged 18 to 70 years old with clear thinking; (3) American Society of Anesthesiologists (ASA) grade I-II; and (4) voluntary participation with ethical support. The exclusion criteria were: (1) emergency surgery; (2) repeat or multiple robot-assisted surgeries; (3) communication or language barriers; and (4) mental disorders or inability to cooperate. The exclusion criteria during the study were: (1) serious violations of the protocol (to be decided by the principal investigator); (2) intraoperative changes in surgical approach, including switching to laparoscopy or open surgery; and (3) occurrence of critical complications such as cardiac arrest, pulmonary embolism, severe arrhythmia, or cerebrovascular accidents during the surgery.

### Grouping program

#### Control group

On the afternoon of the day before surgery, the nurse carrying a tablet computer with the stored robot surgery video visited the patient’s bedside for self-introduction, and then evaluated, educated, provided targeted psychological counseling and support according to the self-made preoperative visit checklist and the prescribed visit process [7]. The robot surgery visit content was added as follows: (1) preoperative assessment of patient’s family income, medical insurance, etc. (2) showing the robot surgery visit video to the patient, including preoperative preparation, precautions, real scene layout of the operating room, anesthesia and coordination, surgical position, the simple process and estimated time about the RPN, entering and returning from the operating room, the process after entering the operating room, characteristics and advantages of robot surgery, Postoperative discomfort, discharge indications, and price department, medical insurance department of robotic surgery charges and reimbursement rate, and the hospital’s robot surgery treatment overview. (3)Video playback in a timely manner to answer the questions raised by the patient, the visit is completed to copy the video into the ward mobile nursing cart.

#### Observation group

(1) Establish a special team for robot surgery: composed of the nurse-in-charge responsible for managing robot surgery and 5 robot surgery nurses, including 1 chief nurse, 3 supervisory nurses, and 2 nurses. Among the 6 members, 3 nurses have received professional training on the da Vinci robot surgery system at the Prince of Wales Hospital in Hong Kong and obtained professional certificates, and 3 nurses have completed the self-organized robot surgery simulation training and passed the assessment [8]. The preoperative visit was undertaken by the members of the special team; (2) Robotic surgery-specific team members were trained on the content of the preoperative visit, the process, guidelines for completing the questionnaire, and evaluation metrics Data collection methodology by the nurse manager; (3) On the afternoon of the day before surgery, the visiting nurse carried a 3D printed model of the kidney to the patient’s bedside for self-introduction, introduced the content, significance and survey questionnaire filling method of the study to the patient and family members. Based on the control group’s preoperative assessment, education, When explaining the procedure, the 3D printed surgical training kidney models was showed. and explaining the location of lesions, size, depth of tumor invasion, the scope of lesions to be resected, and methods of functional reconstruction, combining the features of robotic surgical systems to talk about the advantages of lesion resection and reconstruction, and anatomically induced surgical risks, letting the patients touch the models at the same time as educating and letting the patients watch the video of the surgeon who is going to perform the surgery for them practicing on the models. (4) Quality control: The visit time for each patient was controlled to be around 15 min, leaving 2 to 3 min for nurse-patient interaction.

## Evaluation indicators

### Psychological stress response

The Chinese version of the Amsterdam Preoperative Anxiety and Information Scale (APAIS-C) was used to assess patients’ preoperative anxiety and information needs. The APAIS-C is a self-reported scale that consists of two subscales, anxiety and information needs, with a total of six items. Each item uses a Likert 5-point scoring system, ranging from “completely absent” to “always present.” The anxiety scale score ranges from 4 to 20 points, and the information needs scale score ranges from 2 to 10 points. Higher scores indicate higher levels of preoperative anxiety and information needs. The Cronbach’s α coefficient of the APAIS-C scale in surgical patients is 0.832, and the Cronbach’s α coefficient of the anxiety and information needs subscales is 0.840 and 0.782 [9], respectively. These are all high reliability, and the scale has good test-retest reliability and validity, with test results similar to the original English version. The preoperative assessment was completed by the visiting nurse before the anesthesia induction, and the patients independently filled out the questionnaire. If there were any difficulties, the nurse would read each item aloud without any indication, and the patient would make an independent choice. A total of 80 questionnaires were distributed, and 80 valid questionnaires were collected, with a valid recovery rate of 100%.

### Physiological stress response

The physiological stress of the two groups of patients was evaluated by measuring the levels of serum interleukin 6 (IL-6), angiotensin II (ATII), adrenocorticotropic hormone (ACTH), cortisol (Cor), mean arterial pressure (MAP), and heart rate (HR) at different time points: 6:00 am on the day before surgery (T0), 6:00 am on the day of the operation (T1), 6:00 am on the first day after the operation (T2), and 6:00 am on the third day after the operation (T3). Four milliliters of venous blood were collected on an empty stomach and sent to the laboratory within 30 min. After low-speed centrifugation, the serum levels of ACTH, IL-6, ATII, and cortisol were measured. The patient’s MAP and HR were measured simultaneously.

### Surgical knowledge and preoperative preparation completeness rate

The patients’ surgical knowledge was evaluated using a “Surgical Knowledge Test” designed by the robotic surgery team. The team reviewed the literature and combined it with clinical practice to develop the test, which includes four dimensions: surgical site and name, anesthesia method, preoperative preparation for surgery and anesthesia, robotic surgery-related knowledge, and postoperative complications and prevention, with a total of 24 items. Each item is rated on a Likert 5-point scale, from “completely unaware” to “completely aware,” with scores ranging from 24 to 120 points. Higher scores indicate higher levels of surgical knowledge. The Cronbach’s α coefficient of the test was 0.924, and the content validity index (CVI) was 0.830. The test time, tester, and testing method were the same as those used in the survey of preoperative anxiety and surgical information needs scale. When the patient enters the operating room, the circulating nurse checks the completeness of the preoperative preparation of the patient item by item based on the “Surgical Knowledge Test Scale” in terms of surgical and anesthesia preparation dimensions. If one item is incomplete or incorrect, the patient’s preoperative preparation is considered incomplete. The rate of complete preoperative preparation is calculated as the number of patients with complete preoperative preparation divided by 40 and multiplied by 100%.

### Statistical methods

SPSS 26.0 statistical software was used for data analysis. Count data were described using the number of people and percentages, and intergroup comparisons were made using Pearson chi-square test (χ2) or Fisher’s exact probability method. Normally distributed metric data were expressed as mean ± standard deviation ($\overline {\chi}$± s), and intergroup comparisons were made using independent sample t-test or paired sample t-test. Single-factor analysis of variance (ANOVA) was used for multiple quantitative data, and Bonferroni test was used for pairwise comparisons. A *p*-value < 0.05 was considered statistically significant.

## Results

3.1 Comparison of General Information between the Two Groups (see Table 1). The observation group and control group were compared for general information such as gender, age, education level, marital status, medical expenses payment method, type of surgery, and intraoperative and postoperative use of antihypertensive drugs or not and there were no significant differences (*P* > 0.05), indicating comparability between the two groups.

**Table 1:** Basic information of two groups (*n* = 80)

Group	Quantity	Age($\overline {\chi}$±s)		Gender(Quantity, %)				Degree of education (Quantity, %)							
				Male		Female		Junior high school and below			High school and technical secondary school		College degree and above		
Control group	40	56.08 ± 9.80		25 (62.5)		15 (37.5)		6 (15.0)			25 (62.5)		9 (22.5)		
Observation group	40	54.93 ± 9.62		28 (70.0)		12 (30.0)		8 (20.0)			21 (52.5)		11 (27.5)		
*t*/c^2^		*t*=0.530		χ^2^=0.503				χ^2^=0.834							
*P*		0.598		0.478				0.659							
Group	Marital status (Quantity, %)			Payment method for medical expenses (Quantity, %)				Economic situation (Quantity, %)							
	In marriage	Others		Self funded		Medical insurane/agricultural insurance		Poor			Average		Good		
Control group	30 (75.0)	10 (25.0)		6 (15.0)		25 (62.5)		16 (40.0)			16 (40.0)		8 (20.0)		
Observation group	32 (80.0)	8 (20.0)		8 (20.0 )		21 (52.5)		18 (45.0)			13 (32.5)		9 (22.5)		
χ^2^	0.287			0.834				0.487							
*P*	0.592			0.659				0.784							
Group	Intraoperative and postoperative use of antihypertensive drugs or not (Quantity, %)														
	Yes			No											
Control group	16 (40.0)			24 (60.0)											
Observation group	18 (45.0)			22 (55.0)											
χ^2^	0.205														
*P*	0.651														

"Others" in marital status includes unmarried, divorced, or widowed

3.2 Comparison of psychological stress indicators between the two groups before preoperative visit and anesthesia induction (see Table 2). Before the preoperative visit, there was no statistically significant difference in anxiety and surgery information demand scores between the two groups (*P* = 0.897, 0.737, respectively); before anesthesia induction, the anxiety and surgery information demand scores in the observation group were lower than those in the control group, with statistically significant differences (*P* < 0.001); the anxiety and surgery information demand scores in both groups before anesthesia induction were lower than those before the preoperative visit, and the differences were statistically significant (*P* < 0.05).

**Table 2:** Comparison of anxiety and surgical information needs scores between two groups before visit and anesthesia induction((*n* = 80,$\overline {\chi}$±s)

Group	Quantity	Anxiety score		t	*P*	Scoring of surgical information needs		t	*P*
		Before Visit	Before anesthesia induction			Before Visit	Before anesthesia induction		
Control group	40	13.45± 2.46	12.98 ± 2.28	2.829	0.007	7.23 ± 1.33	7.08 ± 1.29	2.223	0.032
Observation group	40	13.53± 2.72	7.98 ± 1.39	18.025	< 0.001	7.13 ± 1.32	4.03 ± 1.27	17.045	< 0.001
*t*		0.129	11.845			0.337	10.658		
*P*		0.897	< 0.001			0.737	< 0.001		

**Fig. 2:**
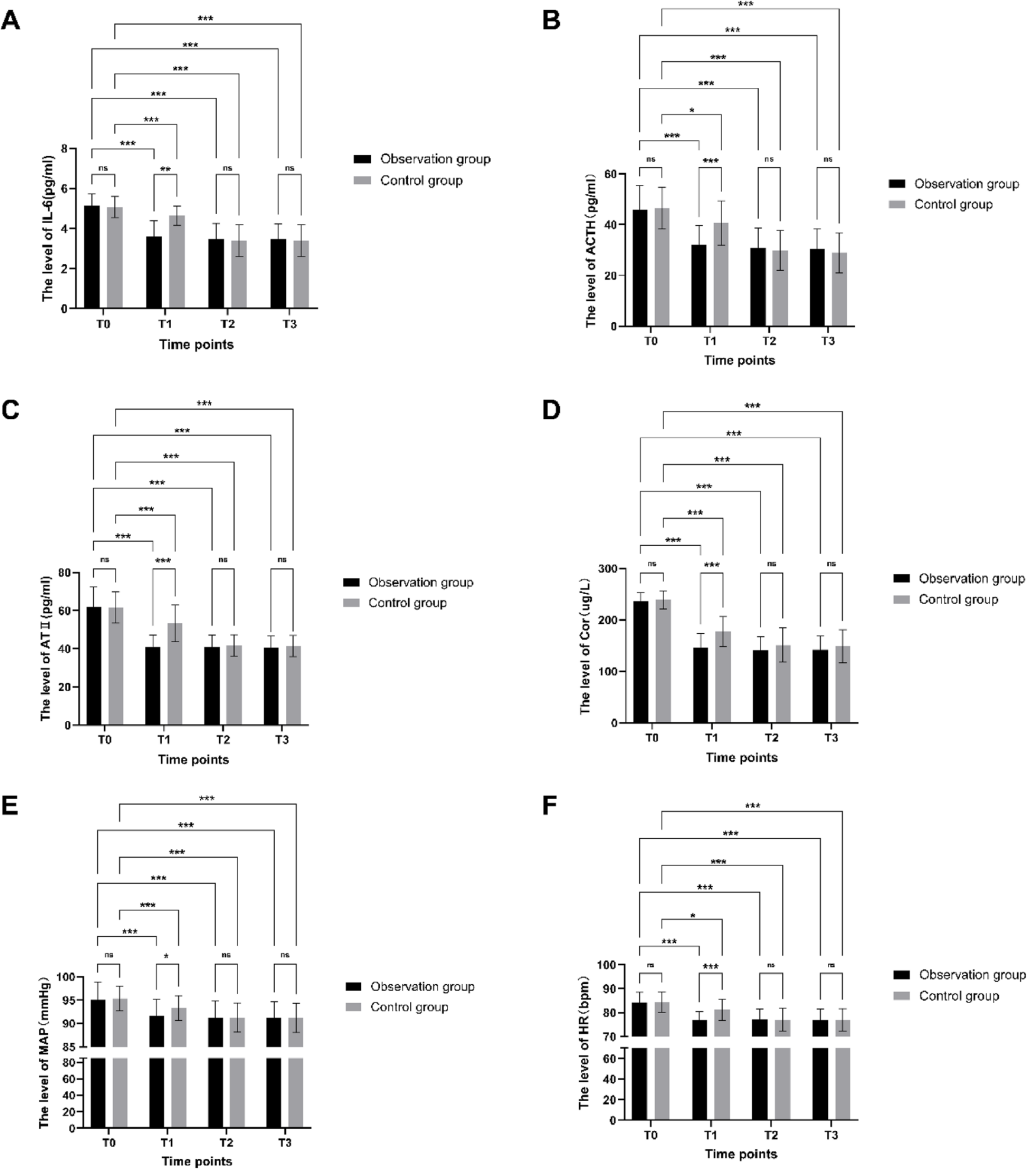
The levelT of physiological stress indicators in different time points between the two groups. **A** The level of IL-6, **B** The level of ACTH, **C** The level of ATII, **D** The level of Cor, **E** The level of MAP, **F** The level of HR. (*p < 0.05, **p<0.01, ***p<0.001)

3.3 Comparison of physiological stress indicators between the two groups (see Fig. 2). At T1 time points, the levels of IL-6, ATII, ACTH, Cor, MAP, and HR in the observation group were all lower than those in the control group, and the differences were statistically significant (*P* < 0.05 for all). At T0, T2, and T3 time points, there were no statistically significant differences in the levels of the various physiological stress indicators between the two groups (*P* > 0.05 for all). The overall mean values of the physiological stress indicators within each group were statistically different (*P* < 0.001 for all). Compared with the T0 time point within each group, the levels of the physiological stress indicators were decreased at the T1, T2, and T3 time points in both groups, and the differences were statistically significant (*P* < 0.05 for all).

3.4 Comparison of surgical awareness and preoperative preparation completeness between the two groups (see Table 3). Before the visit, there was no statistically significant difference in surgical awareness between the two groups (*P* = 0.762). Before anesthesia induction, the awareness of the observation group was better than that of the control group, and the difference was statistically significant (*P* < 0.001). The preoperative preparation completeness rate was 97.50% (39/40) in the observation group and 82.50% (32/40) in the control group, and there was a statistically significant difference between the two groups (*P* = 0.034).

**Table 3:** Comparison of surgical knowledge awareness and preoperative preparation completion rate between two groups(*n* = 80)

Group	Quantity	Pre visit surgical knowledge awareness score (_x±s)	Score of surgical knowledge awareness before anesthesia induction(_x±s)	Preoperative preparation completion rate Quantity(%)
Control group	40	66.75 ± 7.22	77.48 ± 5.79	32(82.50%)
Observation group	40	67.20 ± 5.95	107.75 ± 6.03	39(97.50%)
*t/χ* ^2^		*t* = 0.304	*t* = 22.905	*χ*^2^ = 4.507
*P*		0.762	< 0.001	0.034

## Discussion

### 3D printed surgical training model of the kidney applied to RPN preoperative visit can alleviate patients’ negative emotions

In China, the da Vinci robotic surgery system was introduced in 2006, and the first robotic-assisted laparoscopic open-heart heart surgery was performed in January of the following year [10], so the development of robotic surgery in China is relatively late, and some patients do not have enough access to information related to robotic surgical characteristics and therapeutic effects, Westborg et al [11] research finding that patients who do not receive sufficient information before surgery may have doubts about the surgery, increasing their preoperative anxiety and anxiety. 3D printed surgical model can accurately reflect the internal structure of tissues or organs, more intuitively reflect the location of the patient’s lesions, size, depth of invasion and the surrounding vascular information, image vividly show the spatial anatomical relationship of the lesions, is conducive to patients and their families have a more intuitive in-depth understanding of the disease, the surgery, through the patient’s texture is closer to the normal organization of the 3D printing model touch, so that patients have an additional sense of reality, and at the same time, patients have a sense of realism, so that the patient additional sense of reality, at the same time, touch stimulates the skin tactile receptors, through tiny current signals transmitted to the brain along with the nerve fibers, regulating the vestibular nucleus response to tactile stimulation, so that the organism tactile nerves and the brain to process the information parts of the effective connection, to further satisfy the patient’s need for surgical information [12]. Showing the scene of repeated training of doctors not only avoids bad stimulation with surgical videotapes of actual patients, but also shows the repeated training videos of doctors, so that patients can trust the surgical services based on these systematic trainings more, and increase their trust in the surgical team. The results of this study show that after the preoperative visit, patients in the observation group avoided negative emotions such as anxiety, fear and nervousness caused by exaggerated awareness of surgery and surgical tools risks due to lack of correct knowledge. The anxiety and information need scores before induction of anaesthesia were significantly lower than those before the visit, and the anxiety and information need scores before induction of anaesthesia were significantly lower than those of the control group, with statistically significant differences (*P* < 0.001).

### 3D printed surgical training model of the kidney applied to RPN preoperative visit can alleviate patients’ negative emotions

When patients are in a state of psychological stress, the body produces a series of protective responses, known as stress responses. Moderate stress responses are beneficial to the body, while excessive stress responses increase the risk of intraoperative and postoperative complications [13]. Stress responses mainly affect the body’s immune function through the neuroendocrine pathway, such as the secretion and release of endogenous opioid substances, hypothalamic-pituitary-adrenal axis hormones, and catecholamines, which suppress cellular and humoral immunity. Surgical trauma stress directly affects the body’s immune function [14]. On the other hand, stress stimuli such as tension, anxiety, and surgery stimulate macrophages and endothelial cells to produce inflammatory cytokines such as interleukin-6 (IL-6). IL-6 participates in the synthesis of numerous inflammatory factors, accelerates the inflammatory response, and exacerbates bodily damage [15]. Psychological stress causes excessive activity in the central nervous system and sympathetic nervous system, promoting the elevation of adrenocorticotropic hormone (ACTH), cortisol, and angiotensin II (ATII), leading to loss of appetite, sleep disorders, increased blood pressure, increased heart rate, and an increased risk of surgical complications, which affect the prognosis [14, 16]. In this study, the physiological stress indicators of IL-6, ATII, ACTH, cortisol, mean arterial pressure (MAP), and heart rate (HR) in both groups of patients increased and accelerated at the T0 time point. After the visit, at the T1, T2, and T3 time points, the physiological stress indicators of both groups of patients decreased and slowed down compared to T0, with statistical significance (*P* < 0.05). After the T2 time point, the physiological indicators of both groups tended to stabilize. The comparison between the two groups showed that the changes in physiological stress indicators at the T1 time points were statistically significant, further indicating that when preoperative educational information meets patient expectations, it can effectively alleviate the psychological and physiological stress responses of patients.

### 3D printed surgical training model of the kidney applied to RPN preoperative visit improves patients’ awareness of surgery and preoperative preparation perfection rate

China’s preoperative visit after decades of exploration has achieved certain results, but there is still a missionary process of patients and their families can not understand the spatial location of human organs, especially patients and families without a medical background, 3D printing model can present intuitive realistic surgical effects, to facilitate the different levels of surgical patients and their families to better understand and grasp the content of the missionary to improve the cognitive awareness of the disease, the operation program [17, 18] and compliance with medical and nursing measures. This study shows that the awareness of disease and surgery and the rate of perfect preoperative preparation in the observation group were significantly higher than that in the control group after the visit, and the differences were all statistically significant (all *P* < 0.05).

Through a preoperative visit mode of verbal education, video watching, 3D printing of surgical training models, and tactile explanations of the models, this study improved the patients’ awareness of the surgery, surgical tools, and anesthesia risks, increasing their sense of safety and alleviating negative emotions. This approach effectively reduced psychological and physiological stress, making patients more willing to cooperate with surgical treatment and nursing care, improving the preoperative preparation rate, ensuring the smooth implementation of surgical treatment, and improving the quality of nursing work in the operating room.The 3D printing models used for education are training models of our hospital’s surgical physicians. They are easily accessible and do not add to the department’s expenses. Furthermore, they have great potential for widespread application and promotion.The study focused on robot-assisted partial nephrectomy and was limited to a single center with a small sample size, so future research is needed to verify the effectiveness of this approach in a larger sample size across multiple centers and disease types.

## Data Availability

The datasets generated or analyzed during this study areavailable from the corresponding author on reasonablerequest.
